# Retraction Note: Decreased expression of miR-939 contributes to chemoresistance and metastasis of gastric cancer via dysregulation of SLC34A2 and Raf/MEK/ERK pathway

**DOI:** 10.1186/s12943-022-01702-w

**Published:** 2022-12-28

**Authors:** Jia-Xing Zhang, Yi Xu, Ying Gao, Cui Chen, Zhou-San Zheng, Miao Yun, Hui-Wen Weng, Dan Xie, Sheng Ye

**Affiliations:** 1 Department of Oncology, the First Affiliated Hospital, Sun Yat-sen University, No. 58, Zhongshan road II, 510080 Guangzhou, People’s Republic of China; 2 The State Key Laboratory of Oncology in South China, Sun Yat-Sen University Cancer Center, Collaborative Innovation Center for Cancer Medicine, No. 651, Dongfeng Road East, 510060 Guangzhou, People’s Republic of China; 3 Department of Pathology, Sun Yat-Sen University Cancer Center, Guangzhou, People’s Republic of China

**Retraction Note: Mol Cancer 16, 18 (2017)**

**https://doi.org/10.1186/s12943-017-0586-y**

The Editor-in-Chief has retracted this article. After publication, the authors requested corrections to Figs. 1F, 3A and 3C due to image misuse. Subsequently, concerns were raised regarding similarity between Fig. 1F (top left panel) in this article and Fig. 3C (bottom right panel) in another article [1], as well as image overlap in the top left images of Fig. 3B and 3C.

Further checks by the Publisher have identified the following:

- In Fig. 2C, the flow cytometry analysis for SGC-7901 cells uses inconsistent gating (control group different from all other groups).
- In Fig. 5G, there appears to be high similarity between the p-AKT western blot images (SGC-7901 lane 4 and MKN-45 lane 4, flipped horizontally).

The authors have provided raw data to address these concerns. However, the data contain further discrepancies. The Editor-in-Chief therefore no longer has confidence in the presented data.

Dan Xie has not responded to any correspondence from the editor or publisher about this retraction. Sheng Ye has stated on behalf of the remaining co-authors that they disagree with this retraction.
